# Relationships between social isolation, neighborhood poverty, and cancer mortality in a population-based study of US adults

**DOI:** 10.1371/journal.pone.0173370

**Published:** 2017-03-08

**Authors:** Andrea Fleisch Marcus, Alex H. Illescas, Bernadette C. Hohl, Adana A. M. Llanos

**Affiliations:** 1 Department of Nutritional Sciences, Rutgers School of Health Professions, Newark, NJ, United States of America; 2 Department of Epidemiology, Rutgers School of Public Health, Piscataway, NJ, United States of America; 3 Rutgers School of Criminal Justice, Newark, NJ, United States of America; 4 Rutgers Cancer Institute of New Jersey, New Brunswick, NJ, United States of America; Vanderbilt University, UNITED STATES

## Abstract

**Background:**

Social isolation is an important determinant of all-cause mortality, with evidence suggesting an association with cancer-specific mortality as well. In this study, we examined the associations between social isolation and neighborhood poverty (independently and jointly) on cancer mortality in a population-based sample of US adults.

**Methods:**

Using data from the Third National Health and Nutrition Examination Survey (NHANES III; 1988–1994), NHANES III Linked Mortality File (through 2011) and 1990 Census, we estimated the relationship between social isolation and high neighborhood poverty and time-to-cancer death using multivariable-adjusted Cox proportional hazards models. We examined the associations of each factor independently and explored the multiplicative and additive interaction effects on cancer mortality risk and also analyzed these associations by sex.

**Results:**

Among 16 044 US adults with 17–23 years of follow-up, there were 1133 cancer deaths. Social isolation (HR 1.25, 95% CI: 1.01–1.54) and high neighborhood poverty (HR 1.31, 95% CI: 1.08–1.60) were associated with increased risk of cancer mortality adjusting for age, sex, and race/ethnicity; in sex-specific estimates this increase in risk was evident among females only (HR 1.39, 95% CI: 1.04–1.86). These associations were attenuated upon further adjustment for socioeconomic status. There was no evidence of joint effects of social isolation and high neighborhood poverty on cancer mortality overall or in the sex-stratified models.

**Conclusions:**

These findings suggest that social isolation and higher neighborhood poverty are independently associated with increased risk of cancer mortality, although there is no evidence to support our *a priori* hypothesis of a joint effect.

## Introduction

Social isolation (defined as a lack of participation in social relationships and/or a complete or near-complete lack of interaction with others and/or with society at large) is a well-established determinant of all-cause mortality [1–3]. Accumulating evidence has also suggested an association between social isolation and cancer-specific mortality [4–6], as well as an association with poorer prognosis among some cancer sites [6–11]. Conversely, there is evidence that social integration (defined as participation in a broad range of social relationships; the opposite of social isolation in this context) is associated with lower rates of mortality [2]. Recent findings from our group [12] showed that neighborhood poverty is an important predictor of social integration, particularly the type and quantity of social ties which impacts health outcomes, and further highlighted the importance of studying the effects of neighborhood factors in the context of social relationships and their joint effects on health outcomes.

Given the clear evidence supporting the importance of “place” within the context of health [13], neighborhood characteristics, including aspects of the social, economic and physical environment, may provide additional information on the contextual effects of a community on the health of the individual [14–16]. Socioeconomic status (SES) measures (e.g., education, income, employment), both at the individual and area-based levels, have been shown to be associated with health status in general [17–20], and cancer-related mortality in particular [21–23]. While neighborhood SES has been examined in the context of cancer outcomes and studies have shown that residents of areas characterized by lower SES are more likely to experience significantly worse outcomes [14–16, 24, 25], the role these factors play as determinants of poorer cancer outcomes is not clearly understood.

In prior work, we examined the joint contribution of neighborhood poverty and social isolation to all-cause mortality [26], prompting us to wonder whether this effect is unique to specific causes of death. The conceptual framework that guides this line of inquiry is based on the work of Berkman et al. [27], Krieger’s eco-social theory [28], and the assertion by Diez Roux [29] that the relationships between individuals and the group contexts within which they live and experience relationships are dynamic and reciprocal. In the present study, we examined the joint effect of social isolation and neighborhood poverty on cancer-specific mortality in a national sample of US adults with approximately 20 years of follow-up. We hypothesized that there is an increased risk of cancer mortality associated with both social isolation and high neighborhood poverty (independently), and that the joint effect of these factors would increase cancer mortality risk even when controlling for age, race/ethnicity and individual-level SES.

## Materials and methods

### Study design and measures

We used data from the Third National Health and Nutrition Examination Survey (NHANES III), National Death Index (NDI), and 1990 Census for the present analysis. NHANES III[30] employed a complex, multi-stage, stratified sampling design intended to recruit a nationally representative sample of the non-institutionalized, civilian US population and was conducted from October 1988–1994. We used the adult household interview data for respondents that were ≥17 years old. NHANES III Linked Mortality File contains follow-up data for respondents through December 31, 2011, with mortality status assigned using the NDI [31]. Westat geocoded and matched respondents’ home addresses to 1990 Census tracts for the National Center for Health Statistics (NCHS) [32]. We used the 1990 Census file compiled by the Public Health Disparities Geocoding Project at the Harvard School of Public Health [33], which contains a measure of the percent of residents in each census tract living below the federal poverty level (FPL). We used census tracts to represent neighborhoods and measured the proportion of residents in a census tract living below the FPL with a two-level classification based on the federal definition. FPL has been shown to be a reliable measure of area-level SES [21]. We defined low poverty neighborhoods as census tracts where <20% of the residents lived below FPL and high poverty neighborhoods as those where ≥20% of the residents lived below FPL. Race/ethnicity was self-reported as non-Hispanic White (NHW), non-Hispanic Black (NHB), Mexican-American, and other. Individual SES was based on years of education completed (<8, 9–11, 12, and ≥13 years) and household poverty-to-income ratio (<1, 1–1.99, 2–2.99, 3–3.99, and >4) [30].

We used a modified Social Network Index (SNI) to define our measure of social isolation. SNI captures the four domains first assessed by Berkman and Syme [34], and has been used in previous analyses of NHANES III data [35]. We assigned one point for each of the following: married or living as married, >156 contacts with friends and family in the past year, attending ≥4 religious services in past year, and participating in a voluntary organization. The total SNI score ranged from 0–4. In the analysis, we used a dichotomized SNI variable: a score of 0 or 1 indicated ‘social isolation’ (unfavorable) and a score of 2–4 indicated ‘social integration’ (favorable; referent group). This categorization scheme is consistent with that used in previous work [12, 26, 35]. We conducted sensitivity analysis, where SNI was included as an ordinal variable in the initial Cox models, to examine the appropriateness of using this measure in the present analysis. The results were consistent with those of the dichotomized variable.

The full sample of adult NHANES III participants eligible for follow-up was 20 024. We excluded respondents if their addresses were not geocoded (n = 2778) or if they lived in their current neighborhood for <1 year (n = 1202). The final analytic sample consisted of 16 044 respondents or less for analyses where values for included variables are missing. Both the geocoded NHANES III and the linked mortality data were made available for restricted-use through the National Center for Health Statistics Research Data Center to assure confidentiality of the study participants. These data are not able to be made publically available except through that office. The Institutional Review Board at Rutgers University approved the study.

### Statistical analysis

The outcome of interest was time-to-death (person-months of follow-up) due to cancer-related mortality. We identified a death as cancer-related if the underlying cause was one of the following ICD-10 codes: C00-C97. There were 16 044 participants eligible for the linkage who also met the other criteria for inclusion and 1133 cancer deaths occurred during the study period. There were 17–23 years of follow-up for the study sample depending on the year of NHANES interview with a mean of 215 months (95% confidence interval [CI]: 210, 220) for the weighted sample.

Age, sex, race/ethnicity, and individual SES were adjusted for in the multivariable models. As social isolation has been shown to impact the health of men and women differently [34, 36–39], the models were also stratified by sex to more closely examine its effect on cancer-related mortality. We accounted for the complex sample design of NHANES III in all analyses by applying the appropriate weighting, strata and primary sampling unit variables using SUDAAN, version 11. We employed descriptive statistics to summarize the weighted characteristics of the sample. We used Cox proportional hazards regression to model the relationships between social isolation, neighborhood poverty and months to cancer-related death while adjusting for covariates. If no cancer-related death was recorded, we censored at the end of the follow-up period or other death. The proportional hazards assumption was examined using Kaplan-Meier curves. We assumed that death occurred in this sample at a steady rate equal to that in the general US population and that participation did not alter their mortality risk. We considered self-rated health as a covariate under the assumption that people with a cancer diagnosis and who may have been feeling ill at the time of interview may have a level of social isolation that was directly related to their health including possible cancer diagnoses. However, in these models the main effect hazard ratio (HR) did not change and we ultimately excluded self-rated health as a covariate.

We ran stratified models with main effect social isolation by neighborhood poverty to assess multiplicative interaction. We then inserted a four-level dummy variable combining SNI and neighborhood poverty into a joint-effects model to assess additive interaction. The referent category consisted of individuals with the lowest risk (social integration and low neighborhood poverty). The comparison groups were social integration/high neighborhood poverty, social isolation/low neighborhood poverty, and social isolation/high neighborhood poverty (highest risk group). The referent group represents the absence of the main effects, the high risk group represents the joint effects and the other variables represent the independent effects. We used these HRs to calculate measures of additive interaction [40]; however as these results were null they were not presented.

## Results

The demographic characteristics of the overall study sample and by SNI score and neighborhood poverty groups are presented in Table 1. Briefly, 67% of the weighted sample was <50 years and 53% were female. The majority was NHW (74%), earned incomes placing them at or above FPL (87%) and had at least a high school education (89%). Nearly 6% died from a cancer-related cause during the follow-up period. There were few differences in demographic characteristics by SNI, but there was a higher proportion of women experiencing high social integration than those experiencing social isolation (54% vs. 51%, *P*<0.0001). Those classified as socially integrated were more likely to have at least some college education (*P*<0.0001) compared to those classified as socially isolated. Differences by neighborhood poverty were more obvious. There were slightly more women residing in high poverty neighborhoods (i.e., ≥20% of residents living below FPL; [56% vs. 52%, *P*<0.0001]). The proportion of NHBs, Mexican-Americans and those of other race/ethnicity was higher in high poverty neighborhoods (*P*<0.0001). However, the proportion of cancer-related deaths did not differ by neighborhood poverty.

**Table 1:** Weighted demographics characteristics of the study population presented by neighborhood poverty and social network index, NHANES III, N = 16 044.

Age	Total	Social isolation (SNI = 0,1)	Social integration (SNI = 2–4)	Neighborhood poverty < 20%	Neighborhood poverty ≥20%
	% (SE)	% (SE)	% (SE)	% (SE)	% (SE)
17–19	4.76 (0.35)	6.09 (0.44)	3.94 (0.40)	4.46 (0.40)	6.03 (0.51)
20–29	19.80 (0.81)	25.33 (1.15)	15.14 (0.92)	18.64 (0.85)	24.84 (1.55)
30–39	23.73 (0.75)	22.42 (1.09)	23.76 (0.94)	24.00 (0.94)	22.58 (1.33)
40–49	17.86 (0.62)	14.61 (0.82)	20.37 (0.76)	18.41 (0.73)	15.48 (1.06)
50–59	11.91 (0.41)	10.76 (0.66)	13.37 (0.58)	12.43 (0.49)	9.68 (0.63)
60–69	10.89 (0.49)	9.03 (0.54)	12.57 (0.69)	11.05 (0.59)	10.22 (0.74)
70–79	7.63 (0.41)	7.32 (0.50)	8.10 (0.44)	7.68 (0.48)	7.44 (0.62)
≥80	3.41 (0.29)	4.43 (0.34)	2.76 (0.28)	3.33 (0.35)	3.73 (0.39)
**Sex**					
Male	47.11 (0.47)	48.97 (0.80)	46.38 (0.55)	47.92 (0.59)	43.57 (0.92)
Female	52.89 (0.47)	51.03 (0.80)	53.62 (0.55)	52.08 (0.59)	56.43 (0.92)
**Race/ethnicity**					
Non-Hispanic White	73.60 (1.35)	72.76 (1.78)	79.48 (1.09)	80.75 (1.53)	42.42 (2.30)
Non-Hispanic Black	12.29 (0.74)	12.23 (0.78)	9.83 (0.61)	7.41 (0.69)	33.52 (2.07)
Mexican-American	5.62 (0.48)	5.23 (0.46)	4.55 (0.37)	4.02 (0.46)	12.59 (1.06)
Other	8.49 (0.93)	9.78 (1.36)	6.14 (0.66)	7.81 (1.09)	11.48 (1.72)
**Poverty income ratio**					
Below Poverty	12.75 (0.90)	16.52 (1.05)	8.56 (0.69)	8.77 (0.83)	30.62 (1.86)
≥Poverty	87.25 (0.90)	83.48 (1.05)	91.44 (0.69)	91.23 (0.83)	69.38 (1.86)
**Education**					
<High school	11.09 (0.61)	14.34 (0.86)	8.94 (0.55)	8.43 (0.61)	22.67 (1.11)
Some high school	14.76 (0.60)	18.00 (0.81)	12.86 (0.71)	13.17 (0.68)	21.66 (0.90)
High school grad	32.83 (0.83)	36.08 (0.93)	32.84 (0.95)	33.04 (0.99)	31.91 (1.48)
At least some college	41.32 (1.27)	31.58 (1.40)	45.36 (1.47)	45.36 (1.38)	23.76 (1.29)
**Length of residence in current neighborhood**					
Whole life	26.78 (1.14)	27.33 (1.03)	27.73 (1.36)	25.60 (1.28)	31.92 (1.35)
>20 years	26.64 (0.89)	24.33 (1.16)	27.93 (0.87)	26.60 (1.00)	26.83 (1.45)
11–20 years	15.63 (0.72)	14.87 (0.86)	16.21 (0.76)	16.15 (0.83)	13.35 (1.03)
5–10 years	14.62 (0.76)	15.24 (0.95)	13.84 (0.76)	15.24 (0.84)	11.92 (0.97)
3–4 years	7.60 (0.48)	8.29 (0.77)	6.62 (0.58)	7.60 (0.53)	7.61 (1.00)
1–2 years	8.73 (0.59)	9.93 (0.70)	7.67 (0.66)	8.81 (0.57)	8.36 (1.45)

**Fig 1:**
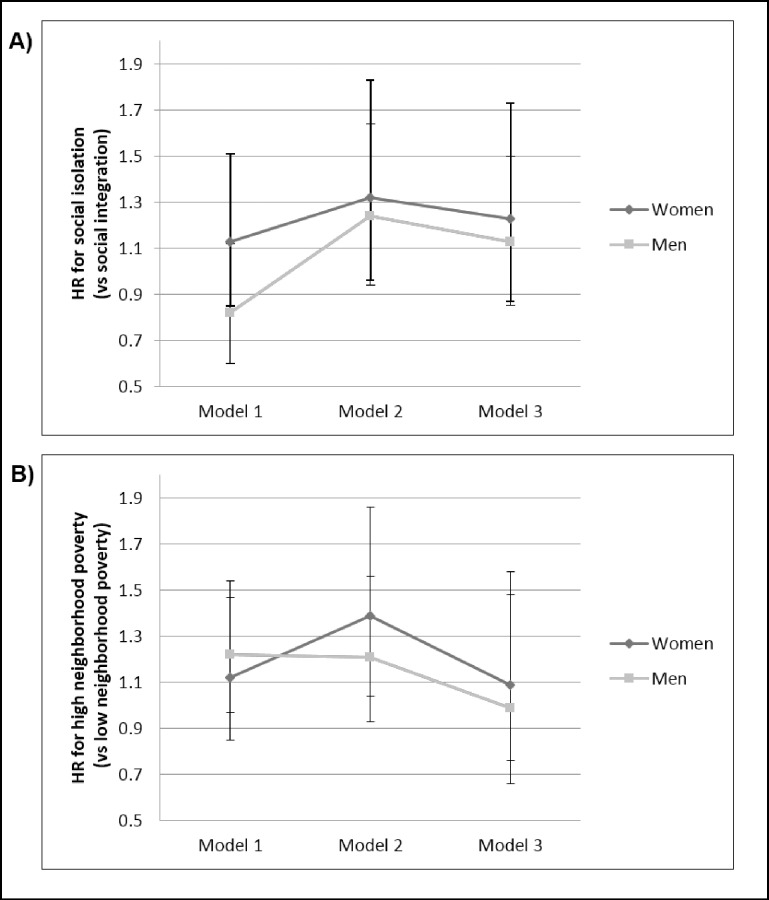
Sex-stratified hazard ratios (HR) of the effects of Social Network Index (A) and Neighborhood Poverty (B) on cancer mortality. In model 1, the HRs are unadjusted, in model 2 they are adjusted for race/ethnicity and age, and in model 3 they are adjusted for race/ethnicity, age, education, and poverty income ratio. The error bars represent the 95% confidence intervals around each HR.

In the unadjusted model (Table 2), social isolation was not associated with cancer mortality (HR 0.97, 95% CI 0.79, 1.20); however after adjusting for age, sex, and race/ethnicity, the risk of cancer mortality was 25% higher among individuals experiencing social isolation compared to those experiencing social integration (HR 1.25, 95% CI: 1.01, 1.54). Upon further adjustment for individual SES (poverty-to-income ratio and education), this association was attenuated (HR 1.15, 95% CI 0.92, 1.43), although the suggestion of increased mortality risk remained evident. No significant association was found between high neighborhood poverty and cancer mortality in the unadjusted model (HR 1.16, 95% CI 0.97, 1.39); however, after adjusting for age, sex, and race/ethnicity, cancer mortality risk was 31% higher among those residing in high poverty neighborhoods (HR 1.31, 95% CI: 1.08, 1.60) than low poverty neighborhoods. This relationship was attenuated in the fully adjusted model (HR 1.04, 95% CI 0.84, 1.33). When the models were stratified by sex, there was slightly higher risks of cancer mortality among women with social isolation (HR 1.32, 95% CI: 0.96, 1.83) and residence in high poverty neighborhoods (HR 1.39, 95% CI: 1.04, 1.86) when adjusted for age and race/ethnicity (Fig 1). Again these associations were attenuated upon further adjustment for individual-level SES.

**Table 2:** Cox proportional hazards models of the associations between social network index and neighborhood poverty with cancer mortality.

Overall sample	Model 1^a^		Model 2^b^		Model 3^c^	
	HR (95% CI)	*P*	HR (95% CI)	*P*	HR (95% CI)	*P*
Social Network Index^*^	N = 15 135	0.791	N = 15 135	**0.039**	N = 13 499	0.207
Social isolation (SNI = 0, 1)	0.97 (0.79, 1.20)		**1.25 (1.01, 1.54)**		1.15 (0.92, 1.43)	
Social integration (SNI = 2–4)	1.00 (referent)		1.00 (referent)		1.00 (referent)	
Neighborhood poverty^**^	N = 16 044	0.093	N = 16 044	**0.007**	N = 14 270	0.73
<20%	1.00 (referent)		1.00 (referent)		1.00 (referent)	
≥20%	1.16 (0.97, 1.39)		**1.31 (1.08, 1.60)**		1.04 (0.84, 1.33)	
**Among males only**						
Social Network Index^*^	N = 6989	0.209	N = 6989	0.118	N = 6278	0.399
Social isolation (SNI = 0, 1)	0.82 (0.60, 1.12)		1.24 (0.94, 1.64)		1.13 (0.85, 1.50)	
Social integration (SNI = 2–4)	1.00 (referent)		1.00 (referent)		1.00 (referent)	
Neighborhood poverty^**^	N = 7444	0.094	N = 7444	0.145	N = 6669	0.947
<20%	1.00 (referent)		1.00 (referent)		1.00 (referent)	
≥20%	1.22 (0.97, 1.54)		1.21 (0.93, 1.56)		0.99 (0.76, 1.30)	
**Among females only**						
Social Network Index^*^	N = 8146	0.401	N = 8146	0.090	N = 7221	0.235
Social isolation (SNI = 0, 1)	1.13 (0.85, 1.51)		1.32 (0.96, 1.83)		1.23 (0.87, 1.73)	
Social integration (SNI = 2–4)	1.00 (referent)		1.00 (referent)		1.00 (referent)	
Neighborhood poverty^**^	N = 8600	0.419	N = 8600	**0.025**	N = 7601	0.630
<20%	1.00 (referent)		1.00 (referent)		1.00 (referent)	
≥20%	1.12 (0.85, 1.47)		**1.39 (1.04, 1.86)**		1.09 (0.76, 1.58)	

NOTE: Unweighted N for each model differs due to some missing data for some covariates.

* These HRs represent proportional hazards models where the only main effect was social network integration (does not include neighborhood poverty)

** These HRs represent proportional hazards models where the only main effect was neighborhood poverty (does not include social network index).

^a^ Unadjusted (crude) model.

^b^ Adjusted for age, sex, and race/ethnicity.

^c^ Adjusted for individual socioeconomic status (poverty income ratio and education).

The hazard ratio for the joint effects model of social isolation and high neighborhood poverty (HR 1.15; 95% CI 0.79, 1.69) did not reflect the hypothesis that the joint effect of these factors would further increase cancer mortality risk (Table 3). This finding was confirmed in the model stratified by neighborhood poverty where the risk associated with social isolation was essentially null among those residing in high poverty neighborhoods (HR 1.06, 95% CI 0.75, 1.51) as well as those residing in low poverty neighborhoods (HR 1.16, 95% CI 0.91, 1.48). This finding suggests that social isolation does not have a synergistic effect on the relationship between neighborhood poverty and cancer mortality. Similarly, in the joint effects models stratified by sex, there was no evidence to support a synergistic effect separately among males or females. Models were tested that examined possible mediation of neighborhood poverty by social isolation but did not reveal evidence of this type of relationship.

**Table 3:** Cox proportional regression models of the joint effects of and social network index and neighborhood poverty on cancer mortality (unweighted N = 13 499)

Overall sample	Social integration		Social isolation		Social isolation by strata of neighborhood poverty	
	(SNI = 2–4)		(SNI = 0,1)			
	HR (95% CI)	*P*	HR (95% CI)	*P*	HR (95% CI)	*P*
Neighborhood poverty <20%	1.00 (referent)		1.17 (0.92, 1.50)	0.202	1.16 (0.91, 1.48)	0.227
Neighborhood poverty ≥20%	1.10 (0.88, 1.38)	0.406	1.15 (0.79, 1.69)	0.451	1.06 (0.75, 1.51)	0.732
**Among males only**						
Neighborhood poverty <20%	1.00 (referent)		1.10 (0.78, 1.55)	0.573	1.08 (0.77, 1.52)	0.631
Neighborhood poverty ≥20%	0.92 (0.63, 1.36)	0.675	1.16 (0.81, 1.66)	0.419	1.35 (0.86, 2.10)	0.184
**Among females only**						
Neighborhood poverty <20%	1.00 (referent)		1.33 (0.91, 1.94)	0.140	1.33 (0.92, 1.92)	0.129
Neighborhood poverty ≥20%	1.31 (0.88, 1.96)	0.175	1.18 (0.65, 2.15)	0.571	0.88 (0.48, 1.62)	0.676

NOTE: Hazard ratios (HR) are adjusted for age, gender, race/ethnicity, individual poverty income ratio, and education.

## Discussion

Several studies have shown that social isolation is associated with poorer chronic disease outcomes and mortality [26, 38, 39, 41–44]. The majority of these studies used measures of social isolation similar in construct to the one that we have used in the present study. In this study, however, we observed a null association between social isolation and cancer mortality, in a multivariable model controlling for age, sex, race/ethnicity, and individual SES. Notably, in a model that controlled for only age, sex, and race/ethnicity (with no adjustment for SES), among individuals with social isolation, there was a 25% increased risk of cancer mortality. Similar to our findings, a meta-analysis of 87 studies [5] demonstrated statistically significant risk reductions in cancer mortality (approximately 15–25%) among cancer patients with high levels of perceived social support and larger social networks. Interestingly, findings from the meta-analysis suggested that the observed risk reductions were weaker among studies that controlled for SES. One explanation for this is that controlling for race/ethnicity as well as SES factors (e.g., income, education) is problematic due to the high correlation among these variables in US populations. Evidence to support this possibility include the *minority poverty* hypothesis, which has consistently shown that disparities exist in high concentrations among low-SES minority populations [22]. While, it was not the aim of this paper to more closely examine race, the in-depth analysis of social isolation according to jointly classified race and SES is warranted and should be pursued in future research. Additionally, these low-SES minority populations may also suffer from low social support or lack of beneficial social networks (i.e., those that are of a high quality and/or confer advantageous health effects) [5, 12, 45]. A recent analysis of NHANES III data demonstrated that while high neighborhood poverty was associated with low general social integration (this same SNI measure), it was also associated with a high number of yearly contacts with neighbors or high within neighborhood social integration [12]. This association further supports the notion that different types of social contacts likely have differential impact on health and cancer mortality in particular.

Another consideration is that our attempt to classify social integration/social isolation may have been flawed. This variable was assessed using a modified index of four domains [34] and may not capture the nature and/or quality of the social networks in which individuals are integrated, which may be of greater importance in the relationship with cancer mortality. Furthermore, the SNI may be an outdated measure that may not provide a sensitive assessment of social isolation or social integration given that modern relationships include intangible interactions (e.g., social media connections), which may differ substantially from interactions deemed important during the development of the SNI (e.g., church membership, group associations)[34]. The cross-sectional nature of our exposure variables, including the components of the SNI, does not take into account the fluctuations in these variables over time or their differential impact on health over time. Another limitation of our analysis is that prevalent cancer and the timing of incident cancer within the sample is unknown. We conducted a sensitivity analysis using self-rated health as a proxy for health at baseline but we understand that this did not fully address the possibility that the impact of baseline social interactions for those with prevalent cancer at the time of assessment may be different from that of those who develop cancer later on in the study. A further limitation could be the inclusion of younger people with lower initial cancer risk in the analytic sample. We believe that their inclusion may have resulted in an attenuation of the effect toward a null association.

This study also observed a null association between neighborhood poverty and cancer mortality, in contrast to our *a priori* hypothesis as well as recent national data [22]. Again, prior to adjustment for individual SES, high neighborhood poverty was associated with a 31% increased risk when compared to low neighborhood poverty. This risk rose to nearly 40% when limited to only women. This may be another artifact of the intersection between race/ethnicity and area-level poverty as mentioned above. Poverty has been shown to be strongly associated with women’s cancers in particular [46] and thus, this may be what we are seeing here and, along with the attenuation after adjustment for individual-level SES, possibly a case of over-adjustment.

Given that neighborhood deprivation may negatively impact general health outcomes, particularly as a result of material deprivation (e.g., limited access to high-quality resources and healthcare) as well as through increased exposure to deleterious psychosocial and behavioral risk, it is thought that neighborhood poverty would also influence components of the cancer continuum; of these, the present study is particularly interested in the effects on cancer survivorship/mortality, which have not been widely studied to date. It is possible that neighborhood factors beyond merely deprivation and/or poverty, such as social and built environment, and other contextual factors, may be associated with cancer survivorship/mortality [13]. Additionally, other factors not considered here, such as access to and utilization of cancer care and screening services and deleterious behaviors associated with cancer incidence and mortality (e.g., smoking, alcohol consumption, poor nutrition, obesity) that are associated with poverty and low SES may be much more important in the context of cancer mortality than neighborhood poverty. Furthermore, this study was limited by the unidimensional measure of neighborhood SES that was used. Although neighborhood poverty is a valid measure of area-level SES [20, 47], other factors that were not accounted for (such as those described above) may play an important role which was not captured here [48]. In addition, there is evidence that neighborhood characteristics as perceived by the individual living within the neighborhood may better predict some health outcomes than those captured by census-level variables alone [18, 49–51]. Additional research to improve our understanding of the independent and interaction effects of neighborhood SES and additional multidimensional factors on cancer outcomes, is an important public health priority.

While we observed no evidence of a significant interaction effect of neighborhood poverty and social isolation on cancer mortality, our findings suggest that social isolation may be of little prognostic importance among residents of high poverty neighborhoods; whereas poverty and/or other related neighborhood-level factors (particularly in disadvantaged neighborhoods) are more important predictors of worse cancer-related outcomes. This finding was confirmed in the model stratified by neighborhood poverty, suggesting that the mortality risk associated with social isolation was null among residents of high poverty neighborhoods. Furthermore, among residents of low poverty neighborhoods, the risk of cancer mortality was slightly lower.

## Conclusion

This analysis of the associations between social isolation, neighborhood poverty, and cancer mortality suggests that higher social isolation and higher neighborhood poverty are each associated with an increased risk of cancer mortality. Although we hypothesized about a joint effect of these social determinants, there was no evidence in our findings to support this notion. Understanding the complex pathways through which social and socio-environmental factors impact health- in particular cancer mortality- is an important step toward understanding cancer disparities. Given the limitations associated with the measurements described herein, the purpose of this study was not to provide a definitive answer on these important issues but to push this question into the literature with the hope it will be examined further and more rigorously in the future.

## References

[1] House JS, Landis KR, Umberson D. Social relationships and health. Science. 1988;241(4865):540–5. 3399889 10.1126/science.3399889

[2] Holt-Lunstad J, Smith TB, Layton JB. Social relationships and mortality risk: a meta-analytic review. PLoS Med. 2010;7(7):e1000316 10.1371/journal.pmed.1000316 20668659 PMC2910600

[3] Steptoe A, Shankar A, Demakakos P, Wardle J. Social isolation, loneliness, and all-cause mortality in older men and women. Proc Natl Acad Sci U S A. 2013;110(15):5797–801. 10.1073/pnas.1219686110 23530191 PMC3625264

[4] Beasley JM, Newcomb PA, Trentham-Dietz A, Hampton JM, Ceballos RM, Titus-Ernstoff L, et al Social networks and survival after breast cancer diagnosis. J Cancer Surviv. 2010;4(4):372–80. 10.1007/s11764-010-0139-5 20652435 PMC2978785

[5] Pinquart M, Duberstein PR. Associations of social networks with cancer mortality: a meta-analysis. Crit Rev Oncol Hematol. 2010;75(2):122–37. 10.1016/j.critrevonc.2009.06.003 19604706 PMC2910231

[6] Ikeda A, Kawachi I, Iso H, Iwasaki M, Inoue M, Tsugane S. Social support and cancer incidence and mortality: the JPHC study cohort II. Cancer Causes Control. 2013;24(5):847–60. 10.1007/s10552-013-0147-7 23549959

[7] Hinzey A, Gaudier-Diaz MM, Lustberg MB, DeVries AC. Breast cancer and social environment: getting by with a little help from our friends. Breast Cancer Res. 2016;18(1):54 10.1186/s13058-016-0700-x 27225892 PMC4881170

[8] Kroenke CH, Kubzansky LD, Schernhammer ES, Holmes MD, Kawachi I. Social networks, social support, and survival after breast cancer diagnosis. J Clin Oncol. 2006;24(7):1105–11. 10.1200/JCO.2005.04.2846 16505430

[9] Lutgendorf SK, De Geest K, Bender D, Ahmed A, Goodheart MJ, Dahmoush L, et al Social influences on clinical outcomes of patients with ovarian cancer. J Clin Oncol. 2012;30(23):2885–90. 10.1200/JCO.2011.39.4411 22802321 PMC3410403

[10] Kroenke CH, Kwan ML, Neugut AI, Ergas IJ, Wright JD, Caan BJ, et al Social networks, social support mechanisms, and quality of life after breast cancer diagnosis. Breast Cancer Res Treat. 2013;139(2):515–27. 10.1007/s10549-013-2477-2 23657404 PMC3906043

[11] Kroenke CH, Quesenberry C, Kwan ML, Sweeney C, Castillo A, Caan BJ. Social networks, social support, and burden in relationships, and mortality after breast cancer diagnosis in the Life After Breast Cancer Epidemiology (LACE) study. Breast Cancer Res Treat. 2013;137(1):261–71. 10.1007/s10549-012-2253-8 23143212 PMC4019377

[12] Marcus AF, Echeverria SE, Holland BK, Abraido-Lanza AF, Passannante MR. How Neighborhood Poverty Structures Types and Levels of Social Integration. Am J Community Psychol. 2015;56(1–2):134–44. 10.1007/s10464-015-9732-0 26076667

[13] Gomez SL, Shariff-Marco S, DeRouen M, Keegan TH, Yen IH, Mujahid M, et al The impact of neighborhood social and built environment factors across the cancer continuum: Current research, methodological considerations, and future directions. Cancer. 2015;121(14):2314–30. 10.1002/cncr.29345 25847484 PMC4490083

[14] Bradley CJ, Given CW, Roberts C. Race, socioeconomic status, and breast cancer treatment and survival. J Natl Cancer Inst. 2002;94(7):490–6. 11929949 10.1093/jnci/94.7.490

[15] Tian N, Goovaerts P, Zhan FB, Chow TE, Wilson JG. Identifying risk factors for disparities in breast cancer mortality among African-American and Hispanic women. Womens Health Issues. 2012;22(3):e267–76. 10.1016/j.whi.2011.11.007 22265181 PMC4338013

[16] Whitman S, Orsi J, Hurlbert M. The racial disparity in breast cancer mortality in the 25 largest cities in the United States. Cancer Epidemiol. 2012;36(2):e147–51. 10.1016/j.canep.2011.10.012 22443886

[17] Galea S, Tracy M, Hoggatt KJ, Dimaggio C, Karpati A. Estimated deaths attributable to social factors in the United States. Am J Public Health. 2011;101(8):1456–65. 10.2105/AJPH.2010.300086 21680937 PMC3134519

[18] Schulz AJ, Mentz G, Lachance L, Johnson J, Gaines C, Israel BA. Associations between socioeconomic status and allostatic load: effects of neighborhood poverty and tests of mediating pathways. Am J Public Health. 2012;102(9):1706–14. 10.2105/AJPH.2011.300412 22873478 PMC3416053

[19] Krieger N, Chen JT, Waterman PD, Rehkopf DH, Subramanian SV. Race/ethnicity, gender, and monitoring socioeconomic gradients in health: a comparison of area-based socioeconomic measures—the public health disparities geocoding project. Am J Public Health. 2003;93(10):1655–71. 14534218 10.2105/ajph.93.10.1655 PMC1448030

[20] Subramanian SV, Chen JT, Rehkopf DH, Waterman PD, Krieger N. Racial disparities in context: a multilevel analysis of neighborhood variations in poverty and excess mortality among black populations in Massachusetts. Am J Public Health. 2005;95(2):260–5. 10.2105/AJPH.2003.034132 15671462 PMC1449164

[21] Krieger N, Chen JT, Waterman PD, Soobader MJ, Subramanian SV, Carson R. Geocoding and monitoring of US socioeconomic inequalities in mortality and cancer incidence: does the choice of area-based measure and geographic level matter?: the Public Health Disparities Geocoding Project. Am J Epidemiol. 2002;156(5):471–82. 12196317 10.1093/aje/kwf068

[22] Kish JK, Yu M, Percy-Laurry A, Altekruse SF. Racial and ethnic disparities in cancer survival by neighborhood socioeconomic status in Surveillance, Epidemiology, and End Results (SEER) Registries. J Natl Cancer Inst Monogr. 2014;2014(49):236–43. 10.1093/jncimonographs/lgu020 25417237 PMC4841168

[23] Warner ET, Tamimi RM, Hughes ME, Ottesen RA, Wong YN, Edge SB, et al Racial and Ethnic Differences in Breast Cancer Survival: Mediating Effect of Tumor Characteristics and Sociodemographic and Treatment Factors. J Clin Oncol. 2015. 10.1200/JCO.2014.57.1349 PMC4486344 25964252

[24] Markossian TW, Hines RB, Bayakly R. Geographic and Racial Disparities in Breast Cancer-Related Outcomes in Georgia. Health Serv Res. 2013. 10.1111/1475-6773.12096 PMC3976183 23909950

[25] Turesky RJ, Freeman JP, Holland RD, Nestorick DM, Miller DW, Ratnasinghe DL, et al Identification of aminobiphenyl derivatives in commercial hair dyes. Chem Res Toxicol. 2003;16(9):1162–73. 10.1021/tx030029r 12971805

[26] Marcus AF, Echeverria SE, Holland BK, Abraido-Lanza AF, Passannante MR. The joint contribution of neighborhood poverty and social integration to mortality risk in the United States. Ann Epidemiol. 2016;26(4):261–6. 10.1016/j.annepidem.2016.02.006 27016951

[27] Berkman LF, Glass T, Brissette I, Seeman TE. From social integration to health: Durkheim in the new millennium. Social Sci Med. 2000;51(6):843–57. 10.1016/s0277-9536(00)00065-4 10972429

[28] Krieger N. Methods for the scientific study of discrimination and health: an ecosocial approach. Am J Public Health. 2012;102(5):936–44. 10.2105/AJPH.2011.300544 22420803 PMC3484783

[29] Diez Roux AV. Next steps in understanding the multilevel determinants of health. J Epidemiol Community Health. 2008;62(11):957–9. 10.1136/jech.2007.064311 18854498

[30] National Center for Health Statistics. Third National Health and Nutrition Examination Survey, 1988–1994, NHANES III Household Adult Data File and Documentation. In: Services UDoHaH, editor. Hyattsville, MD: Centers for Disease Control and Prevention; 1996.

[31] National Center for Health Statistics. The Third National Health and Examination Survey (NHANES III) Linked Mortality File, Mortality Follow-up through 2011: Matching Methodology. In: US Department of Health and Human Services, editor. Hyattsville, MD2013.

[32] National Center for Health Statistics. National Health and Nutrition Examination Survey (1988–1994) Documention, Codebook, and Frequencies: Geocoding (N3_GEO) In: US Department of Health and Human Services, editor. Hyattsville, MD2009.

[33] The Public Health Disparities Geocoding Project. U.S. Census Tract Poverty Data Boston, MA: Harvard School of Public Health; 2004 [January 13, 2017]. Available from: http://www.hsph.harvard.edu/thegeocodingproject/webpage/monograph/povdata.htm.

[34] Berkman LF, Syme SL. Social networks, host resistance, and mortality: a nine-year follow-up study of Alameda County residents. Am J Epidemiol. 1979;109(2):186–204. 425958 10.1093/oxfordjournals.aje.a112674

[35] Ford ES, Loucks EB, Berkman LF. Social integration and concentrations of C-reactive protein among US adults. Ann Epidemiol. 2006;16(2):78–84. 10.1016/j.annepidem.2005.08.005 16271297

[36] Kawachi I, Kennedy BP, Lochner K, Prothrow-Stith D. Social capital, income inequality, and mortality. Am J Public Health. 1997;87(9):1491–8. 9314802 10.2105/ajph.87.9.1491 PMC1380975

[37] Kawachi I, Colditz GA, Ascherio A, Rimm EB, Giovannucci E, Stampfer MJ, et al A prospective study of social networks in relation to total mortality and cardiovascular disease in men in the USA. J Epidemiol Community Health. 1996;50(3):245–51. 8935453 10.1136/jech.50.3.245 PMC1060278

[38] Seeman TE, Kaplan GA, Knudsen L, Cohen R, Guralnik J. Social network ties and mortality among the elderly in the Alameda County Study. Am J Epidemiol. 1987;126(4):714–23. 3631060 10.1093/oxfordjournals.aje.a114711

[39] Pantell M, Rehkopf D, Jutte D, Syme SL, Balmes J, Adler N. Social isolation: a predictor of mortality comparable to traditional clinical risk factors. Am J Public Health. 2013;103(11):2056–62. 10.2105/AJPH.2013.301261 24028260 PMC3871270

[40] Knol MJ, VanderWeele TJ. Recommendations for presenting analyses of effect modification and interaction. Int J Epidemiol. 2012;41(2):514–20. 10.1093/ije/dyr218 22253321 PMC3324457

[41] Stringhini S, Berkman L, Dugravot A, Ferrie JE, Marmot M, Kivimaki M, et al Socioeconomic status, structural and functional measures of social support, and mortality: The British Whitehall II Cohort Study, 1985–2009. Am J Epidemiol. 2012;175(12):1275–83. 10.1093/aje/kwr461 22534202 PMC3372313

[42] Ertel KA, Glymour MM, Berkman LF. Effects of social integration on preserving memory function in a nationally representative US elderly population. Am J Public Health. 2008;98(7):1215–20. 10.2105/AJPH.2007.113654 18511736 PMC2424091

[43] Nagayoshi M, Everson-Rose SA, Iso H, Mosley TH Jr., Rose KM, Lutsey PL. Social network, social support, and risk of incident stroke: Atherosclerosis Risk in Communities study. Stroke. 2014;45(10):2868–73. 10.1161/STROKEAHA.114.005815 25139878 PMC4201236

[44] Samuel LJ, Dennison Himmelfarb CR, Szklo M, Seeman TE, Echeverria SE, Diez Roux AV. Social engagement and chronic disease risk behaviors: The Multi-Ethnic Study of Atherosclerosis. Prev Med. 2015;71:61–6. 10.1016/j.ypmed.2014.12.008 25524614 PMC4329061

[45] Plickert G, Cote RR, Wellman B. It's not who you know, it's how you know them: who exchanges what with whom? Social Networks. 2007;29(3):25.

[46] Ginsburg O, Bray F, Coleman MP, Vanderpuye V, Eniu A, Kotha SR, et al The global burden of women's cancers: a grand challenge in global health. Lancet. 2016. 10.1016/S0140-6736(16)31392-7 PMC6191029 27814965

[47] Krieger N, Chen JT, Waterman PD, Rehkopf DH, Subramanian SV. Painting a truer picture of US socioeconomic and racial/ethnic health inequalities: the Public Health Disparities Geocoding Project. Am J Public Health. 2005;95(2):312–23. 10.2105/AJPH.2003.032482 15671470 PMC1449172

[48] Sposto R, Keegan TH, Vigen C, Kwan ML, Bernstein L, John EM, et al The Effect of Patient and Contextual Characteristics on Racial/Ethnic Disparity in Breast Cancer Mortality. Cancer Epidemiol Biomarkers Prev. 2016;25(7):1064–72. 10.1158/1055-9965.EPI-15-1326 27197297 PMC4930680

[49] O'Campo P, Salmon C, Burke J. Neighbourhoods and mental well-being: what are the pathways? Health Place. 2009;15(1):56–68. 10.1016/j.healthplace.2008.02.004 18420446

[50] O׳Campo P, Wheaton B, Nisenbaum R, Glazier RH, Dunn JR, Chambers C. The Neighbourhood Effects on Health and Well-being (NEHW) study. Health Place. 2015;31(0):65–74. 25463919 10.1016/j.healthplace.2014.11.001

[51] Schulz AJ, Mentz G, Lachance L, Zenk SN, Johnson J, Stokes C, et al Do observed or perceived characteristics of the neighborhood environment mediate associations between neighborhood poverty and cumulative biological risk? Health Place. 2013;24:147–56. 10.1016/j.healthplace.2013.09.005 24100238 PMC3837295

